# Influence of Glutaraldehyde on the Properties of Agar-Based Films

**DOI:** 10.1021/acsomega.6c07034

**Published:** 2026-10-20

**Authors:** Gian Lucas Pretto, Júlia Daneluz, Wendel Paulo Silvestre, Camila Baldasso

**Affiliations:** Postgraduate Program in Process Engineering and Technologies (PGEPROTEC), 58802 Universidade de Caxias do Sul, Caxias do Sul 95070-560, Rio Grande do Sul, Brazil

## Abstract

Biopolymers have been used as alternatives to fossil-derived polymers. In this context, agar, a polysaccharide extracted from seaweed, has stood out due to its properties in the formulation of polymeric films. In this work, agar was used in combination with glycerol and glutaraldehyde to formulate polymeric films by the casting method with water as solvent. The films produced were characterized regarding their water interactions using swelling, solubility, and contact angle tests. Mechanical and physical properties were determined using a tensile test and thickness measurements. Optical microscopy was used to analyze the surface, and scanning electron microscopy was used to analyze the surface and cross-section of the films. Spectroscopic properties were obtained by Fourier-transform infrared spectroscopy (FTIR) and ultraviolet–visible spectroscopy. Thermal degradation was determined by thermogravimetric analysis (TGA). It was possible to produce polymeric films with a thickness of around 120 μm and mechanical properties like those found on low-density polyethylene, showing tensile strengths of 15–16 MPa. The solubility of the material was 42% and the swelling ranged between 349–555%, depending on the concentration of glutaraldehyde added to the film formulation of films. Adding glutaraldehyde altered the surface characteristics of the films while maintaining the other properties. However, no evidence of cross-linking reactions was found.

## Graphical abstract

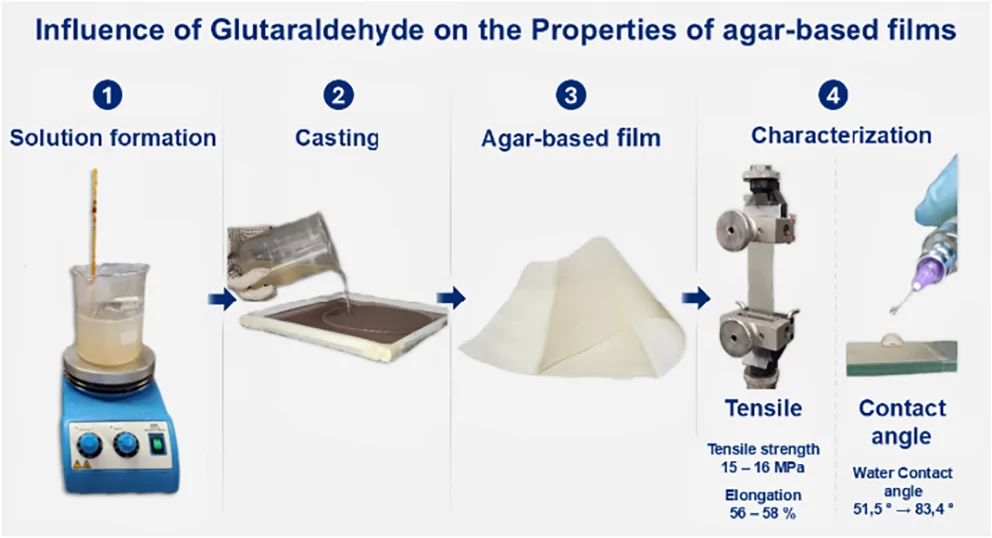

## Introduction

The use of fossil-based polymeric materials in industrial and domestic applications represents one of the biggest contemporary environmental challenges,^1^ especially regarding packaging.^2^ Materials such as polyethylene (PE), polypropylene (PP), and polystyrene (PS) have been widely used for this function, due to their versatility and low cost.^3^

However, its slow degradability in natural environments and the difficulties in reintegrating it into productive cycles (due to its low added value and contamination of packages after use) have promoted the accumulation of waste from these materials and inadequate disposal, causing significant environmental impacts.^4,5^

Interest in the use of materials produced from renewable resources and those that decompose into environmentally friendly substances has grown with the intention of reducing damage to the environment,^6^ particularly with the requirements from governmental guidelines.^7^ Additionally, the scarcity of fossil resources is another motivation for developing biopolymers.^8^ Biopolymers are types of polymers derived from renewable sources, as well as biobased polymers or biodegradable fossil-based polymers.^4^ Biodegradable polymers are, by definition, polymers that are degraded by the actions of microorganisms, such as fungi and bacteria, or that degrade on the ground, resulting in water and carbon dioxide as products.^9^

Biopolymers degrade rapidly and do not release harmful residues, unlike fossil-based polymers. Therefore, the use of biopolymers contributes to environmental conservation, eliminating the need for complex logistics for the collection and reutilization of packaging waste. Due to its rapid decomposition and easier disposal, the impact on the ecosystem is smaller.^4,9^

Biopolymers have been developed from various raw materials, and agar has shown promising properties for their production.^10,11^ The development of agar-based biopolymers has been driven by the aim of producing materials with properties like those of fossil-based polymers. Agar has the potential to be used in biobased film production.^12,13^

Agar is a phycocolloid polysaccharide extracted from red seaweed. Agar, industrially produced, is a light-colored powder.^14^ Due to its natural occurrence as a carbohydrate present on the seaweed cell wall, agar is constituted of a mixture of two polysaccharides: agarose, this being the component responsible for gelation, and agaropectin, the nongelation portion.^10,14^ Agar is a substance with several applications: it can be used in the culinary and food industries as a thickening agent, in the pharmaceutical industry as part of capsules, in medicine formulations, and even in microbiological cultures in research laboratories. This substance stands out for its gelation capability and insolubility in cold water.^14,15^

Polymeric films are prepared from polymers and may contain additives and reinforcement materials.^16^ In polysaccharide-based polymer formulations, additives are commonly used to improve properties and handling characteristics, such as plasticizers and cross-linking agents. Plasticizers such as glycerol, sorbitol, and xylitol increase the polymer’s flexibility,^17–19^ and the cross-linking agent enhances tensile strength and thermal stability while reducing swelling.^20^ The highly versatile nature of some polymer films can be effectively optimized by incorporating appropriate and readily available plasticizers and cross-linking agents, making them suitable for a wide range of practical applications and offering an attractive alternative to the nonenvironmentally friendly materials currently used.^21^

The development of agar-based polymers aims to produce materials with improved properties, such as mechanical resistance and water barrier properties.^16^ This similarity with some polymers enables the use of agar films for food packaging (commonly PP), cosmetics and toys packaging (commonly ABS), and plastic bags (commonly TPS).^12^ In some previous studies, it was observed that increasing the agar ratio reduces solubility, enhances tensile strength and fracture strain, and that increasing the glycerol ratio enhances solubility and reduces tensile strength.^22,23^

Several parameters and additives have been studied to enhance the properties of polymer films for packaging use. Hernández et al. have studied variations in agar and glycerol concentrations to train neural networks and demonstrated that the mechanical properties are compatible with those of some synthetic polymers.^12^ Priyadarshi et al. have used sulfur quantum dots on agar/gelatin blends, which enhanced the films’ tensile strength and provided antimicrobial and anti-UV properties, without altering biocompatibility.^24^ Taminna et al. have used agar on carboxymethylcellulose blends with SnO_2_ and Zn–SnO_2_ particles as additives. They observed an antimicrobial effect, especially with Zn–SnO_2_, while maintaining mechanical properties and improving the UV barrier.^25^ Awadhiya et al. proposed cross-linking agar with citric acid on films, demonstrating enhanced mechanical resistance and reduced swelling.^20^

Although several studies have investigated agar films reinforced with nanoparticles, polymer blends and alternative cross-linking agents such as citric acid, there is still limited understanding of the influence of glutaraldehyde on neat agar films. Most available studies involving glutaraldehyde focus on proteins or synthetic polymers, whereas its effects on agar structure, water interactions, morphology and mechanical behavior remain poorly understood. Furthermore, it is still unclear whether the addition of glutaraldehyde effectively promotes cross-linking in agar matrices under mild casting conditions. Therefore, this work investigates the influence of glutaraldehyde concentration on agar-based films, aiming to determine whether the expected cross-linking reactions translate into measurable changes in physical, mechanical, thermal, spectroscopic and morphological properties.

## Methods

### Materials

Food-grade agar was purchased from Agargel (Brazil), ACS-grade glycerol from Vetec (Brazil), and glutaraldehyde 50 wt % from Dinâmica (Brazil). Distilled water was used in all productions.

### Production of Polymeric Films

Casting solutions of 520 mL having 1.5 wt % agar and 50 wt % glycerol (wt of glycerol/wt of agar) were produced.^20^ The reagents were added to Beakers containing distilled water and heated at 80–87 °C on heating plates for 10 min.^12^ The effects of adding glutaraldehyde were studied by the production of four formulations with concentrations of zero, 5, 10, and 15 wt % (relative to agar mass) of glutaraldehyde (added before heating of the solutions), named AGG0, AGG5, AGG10, and AGG15, respectively. Therefore, to produce this volume of solution 7.80 g of agar and 3.90 g of glycerol were used. Glutaraldehyde 50% wt solution was added in the amounts of zero, 0.78, 1.56, and 2.34 g to each formulation.

After this step, the solutions were immediately cast onto Teflon-coated glass plates with an area of approximately 784 cm^2^ (28 cm × 28 cm, approximately), leveled, bordered with Teflon coated barriers, and preheated to 85 °C in oven.^26^ After cooling, the plates were kept in an environment with controlled temperature and relative humidity of 23 ± 2 °C and 50 ± 10% for 72 h until complete drying.^12,26^ After demolding, the films were maintained in boxes with controlled humidity of 50 ± 10% by saturated solution of Mg­(NO_3_)_2_ and at 15–25 °C until characterization tests were carried out. Each film formulation was produced in triplicates.

### Films Characterization

#### Physical and Mechanical Properties

Film thickness was measured with a dry film thickness gauge (BYK, Byko-test 4500, United States) at ten points distributed over the films.

Tensile test was conducted in accordance with ASTM D882–18 using five rectangular specimens of 120 mm × 20 mm, with a gauge length of 50 mm. Specimen thickness was measured with a micrometer (Mitutoyo, MDC Lite 293–821–30, Japan), and the width was measured with a caliper (Mitutoyo, CD-8″ C, Japan). Specimens were kept at 23 ± 2 °C and a 50 ± 10% relative humidity for 48 h before and during the test. The tensile tests were carried out at a speed of 25 mm·min^–1^ on an universal testing machine (EMIC, DL3000, Brazil) equipped with a 20 kgf load cell for force measurement, and a displacement transducer.

#### Water Interaction

Films’ solubility was determined by the method adapted from Scopel et al.^26^ Three specimens, measuring 20 mm × 20 mm, from each film were cut and kept in desiccator with silica gel for 7 days for initial mass determination. After that, the films were placed in plastic containers of Beaker format containing 70 mL of deionized water and agitated on an orbital shaker (Marconi, MA 832, Brazil) at 90 rpm for 24 h and 25 ± 2 °C.

Specimens were removed from water and kept in a desiccator for 7 days for final mass determination. Mass measurements were made using analytical scale (Shimadzu, AUY220, Philippines). Solubility was calculated by eq 1.

1 $$solubility\;\left(\%\right)=\frac{m_{initial}-m_{final}}{m_{initial}}\cdot 100$$

Film swelling was determined by adapting the method of Awadhiya et al.^20^ Three 20 mm × 20 mm specimens from each film were placed in a desiccator for 72 h. Then, their initial masses were determined on an analytical scale (Shimadzu, AUY220, Philippines). The specimens and 100 mL of deionized water were added to Beakers. After 2.5 h, the samples were removed from immersion, slightly dried with a paper towel, and their final masses were measured. The swelling degree was determined using eq 2.

2 $$swelling\;\left(\%\right)=\frac{m_{final}-m_{initial}}{m_{initial}}\cdot 100$$

The contact angle was determined by the sessile drop method.^27^ A drop of distilled water was placed over the film’s surface. An image was captured with a digital camera (Panasonic, Lumix DMC-FZ40, Japan), and the contact angle was measured with Surftens (OEG GmbH) software.

#### Morphological Properties

Optical microscopy of the surface was performed using a reflected-light microscope (Olympus, BX53M, Japan) at magnifications of 50×, 100×, and 200×.

Scanning electron microscopy (SEM) was performed on microscope (Tescan, MIRA 3 LMU, Czech Republic) at 10 kV, with secondary and backscattered electron capture. Analysis of surface and cross-section (obtained by cryogenic fracturing after sample freezing with liquid nitrogen) was made with magnifications of 250×, 1000×, 5000×, and 10000×.

#### Spectroscopic Properties

Infrared spectroscopy with Fourier transform (FTIR) was carried out using a spectrometer (Thermo Scientific, iS 10, USA) equipped with an ATR accessory. Thirty-two scans were performed at a resolution of 0.5 cm^–1^ over the range of 525–4000 cm^–1^ after 7 days of storage in a desiccator.

Ultraviolet–visible (UV–vis) spectra were obtained using a spectrophotometer (Beckman, DU 530, USA) over the range of 200–800 nm with a 2.0 nm resolution. The films were attached to the cuvette support so that the beam first passed through the surface exposed to air during drying, then through the material.

#### Thermal Properties

Thermogravimetric analysis was carried out in simultaneous thermal analysis instrument (Shimadzu, TGA-50, Japan), using 10 mg of the films previously dried in a desiccator for 7 days. A heating rate of 10 °C·min^–1^ was used with a nitrogen atmosphere (50 mL·min^–1^) and a temperature range of 25–610 °C. The determination of the ranges of the stages of mass loss was made based on the points of inflection showed by the DTG curves.

#### Statistical Treatment

Quantitative data were tested for outliers using the Grubbs test at a 95% confidence level. When detected, outliers were not considered. Statistical differences were determined by Analysis of Variance (ANOVA), followed by the Tukey test for tests with four treatments (AGG0, AGG5, AGG10, and AGG15), and using the Student’s *t* test when comparing two treatments (AGG0 and AGG10), both with a confidence level of 95%.

## Results and Discussion

### Visual Aspect

For all formulations, continuous, translucent, and slightly yellow films were formed. Images of the films are presented in Figure 1.

**Figure 1:**
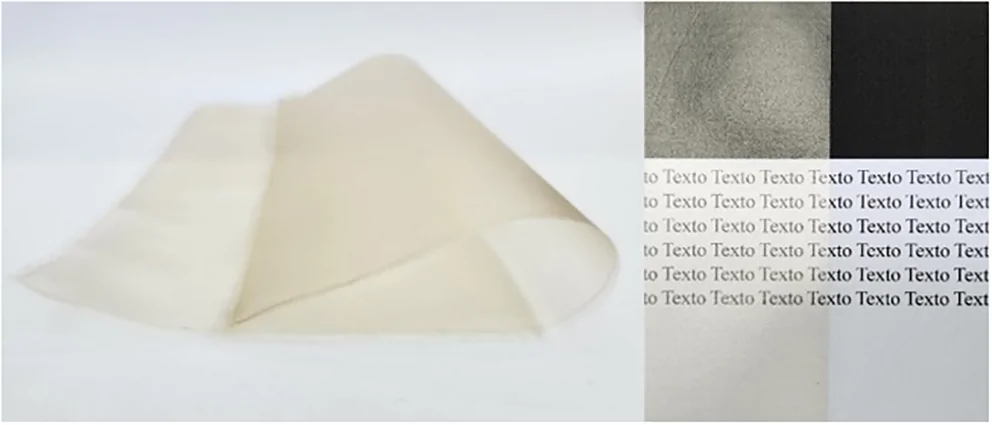
Visual aspects of the films when folded and when displayed over text, black and white background.

### Thickness, Solubility, and Swelling

Thickness, solubility, and swelling properties of the films produced with different ratios of glutaraldehyde are presented in Table 1.

**Table 1:** Thickness, Solubility, and Swelling Properties of Agar Films Produced with Different Ratios of Glutaraldehyde^a^

Formulation	Thickness (μm)	Solubility in water (wt %)	Swelling degree (%)
AGG0	119 ± 5^a^	42.2 ± 2.4^a^	349 ± 15^a^
AGG5	121 ± 6^ab^	41.9 ± 2.3^a^	394 ± 18^ab^
AGG10	124 ± 7^b^	41.8 ± 2.9^a^	476 ± 53^b^
AGG15	125 ± 7^b^	43.7 ± 2.8^a^	555 ± 43^b^

a Data presented as mean ± standard deviation. Different letters in the same column indicate statistically different means (*p*-value < 0.05).

Statistically significant differences in film thickness are observed between formulations with glutaraldehyde (AGG10 and AGG15) and those without it (AGG0). It is possible to establish a tendency of enhancement of thickness in function of the enhancement of the glutaraldehyde ratio. This effect can be explained by the increase in film volume due to the addition of glutaraldehyde in a constant casting area, leaving thickness as the only dimension that can be adjusted with the increase in volume.

The addition of glutaraldehyde at concentrations of 5 wt % and 15 wt % (relative to the agar mass) did not affect the solubility of the films. This effect may indicate that there were no changes in bond strength between the polymer molecules, since the portion of the polymer that detaches and is solubilized is the same in both cases.

In the swelling test, a significant difference was observed between the films with 10 wt % glutaraldehyde (relative to the agar mass), AGG10 and AGG15, and the film without glutaraldehyde (AGG0). A tendency toward increased swelling with increasing glutaraldehyde ratio can also be observed. This effect may be related to the affinity of this substance for water, which may have led to greater water binding to glutaraldehyde at higher concentrations, making the polymer more hydrophilic.

The increase in swelling degree with increasing glutaraldehyde concentration, along with the formulations’ similar solubility behavior, suggests that glutaraldehyde was not fully incorporated into the films’ microstructure. As reported by Bigi et al.,^28^ cross-linking of gelatin films with glutaraldehyde should enhance the barrier to water, thereby decreasing their swelling at higher glutaraldehyde ratios.

Yeom and Lee^29^ reported the effect of excess glutaraldehyde on polymeric films made of poly­(vinyl alcohol). After reaching the maximum possible cross-linking point, the excess of glutaraldehyde not linked to the polymeric chains has its aldehyde groups available to make hydrogen bonds. As a result, glutaraldehyde molecules not covalently linked to polymeric chains can enhance the films’ swelling properties.

The results of swelling can be compared to those found by Isik et al.^30^ that reported values of 325–423% for films made of agar and gelatin, with addition of different concentrations of *Aloe vera* oil (although water immersion period was 24 h). The study also reported an increase in swelling degree as the ratio of *A. vera* oil was increased.

Based on the swelling test results, the AGG10 formulation was considered to have better potential for packaging applications. Therefore, its complete characterization was performed and compared with that of the control sample (AGG0). AGG10 represented the lowest concentration that produced statistically significant changes in swelling without further increases at 15 wt %. Since AGG15 did not provide additional functional advantages, AGG10 was selected as the most representative formulation, also minimizing additive consumption and processing costs.

### Contact Angle

The contact angles with water for the films produced are presented in Table 2.

**Table 2:** Results of Contact Angle with Water of the Films without Glutaraldehyde and with 10 wt % Glutaraldehyde (Relative to the Agar Mass)^a^

Formulation	Contact angle with water (°)
AGG0	51.5 ± 6.6^b^
AGG10	83.4 ± 9.2^a^

a Data presented as mean ± standard deviation. Different letters in the same column indicate statistically different means (*p*-value < 0.05).

The contact angles with water for the tested formulations are shown in Figure 2.

**Figure 2:**
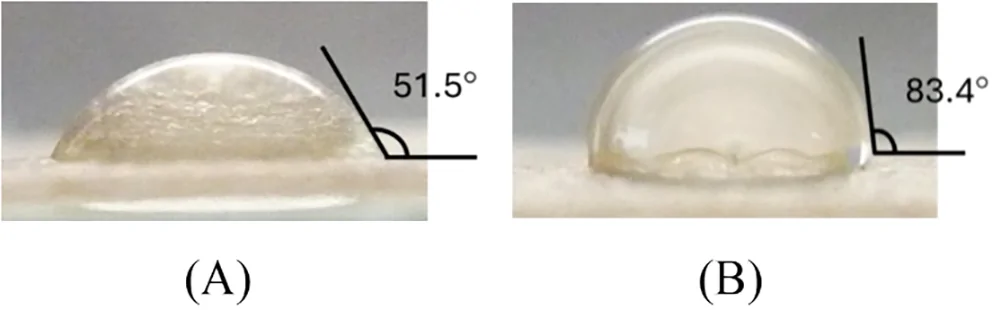
Contact angle with water, in which a drop of distilled water was placed over the films AGG0 (A) and AGG10 (B).

According to the contact angles observed, the formulation AGG10 showed a distinct behavior compared with the swelling test, in which the addition of glutaraldehyde made the polymer more hydrophilic. Contact angle values show the opposite behavior since the addition of glutaraldehyde enhanced this property, rendering the films more hydrophobic. The results for contact angle of formulation AGG10 are like those found by El-Hefian et al.,^31^ that have found values of 88° for chitosan films, and values of around 80° with the addition of 25% wt of agar and PVA.

This difference may be because of glutaraldehyde on the structure of the polymer. Since glutaraldehyde interacts with agar chains, it may cause changes in the roughness of the material, turning its surface more hydrophobic. Superficial cross-linking reactions may also have had some influence on this property, especially on the film’s surface, although this result alone may not confirm the occurrence of this kind of reaction. Considering that the cross-linking mechanisms would be similar to those found in starch and reported by Gönenç and Us,^32^ agar cross-linking would consume some hydroxyl groups on its chains, decreasing the material’s affinity for water and making its surface more hydrophobic, without changing the swelling properties of the films.

It is important to note that the contact angle varies with the intermolecular interactions between polymeric chains and their constituents. Still, it is also influenced by surface characteristics such as roughness. Therefore, changes in contact angle may be due to superficial effects that were altered by the addition of a cross-linking agent.

### Optical Microscopy (OM) and Scanning Electron Microscopy (SEM)

The images of optical microscopy with magnifications of 50×, 100×, and 200× are presented in Figure 3.

**Figure 3:**
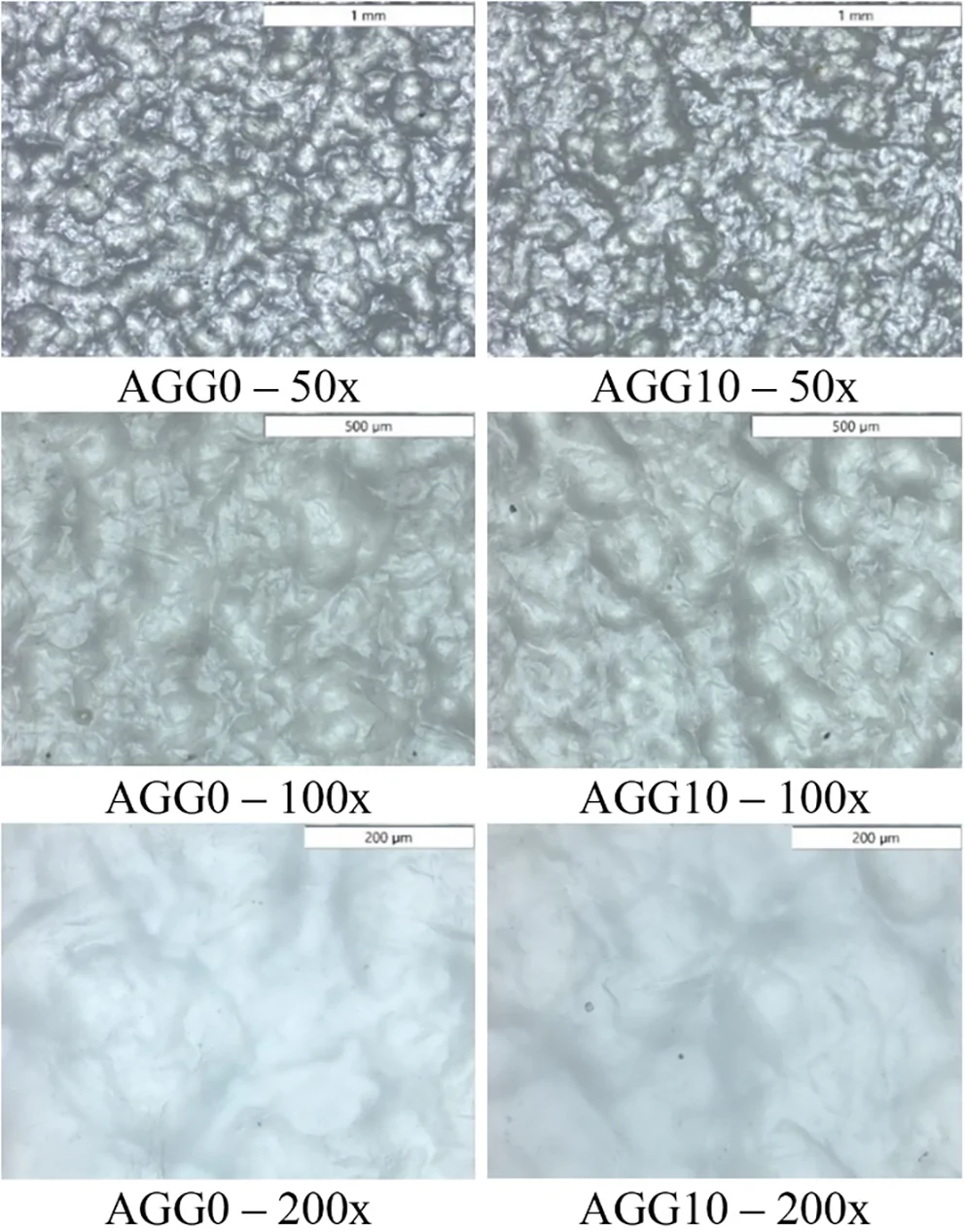
Optical microscopy images of the surfaces of films AGG0 and AGG10.

The surfaces of the films produced with both formulations exhibit nodular or venous structures, making the films appear rough. A factor that may have contributed to this morphology is the drying process. During solvent evaporation, the film contracts, occupying less area than the casting solution did right after gelation. This contraction may be responsible for the formation of roughness, leading to material accumulation in uneven layers along the film, creating regions of peaks and valleys.

SEM images of the surface of films AGG0 and AGG10, with magnifications of 250×, 1000×, and 5000×, are shown in Figure 4.

**Figure 4:**
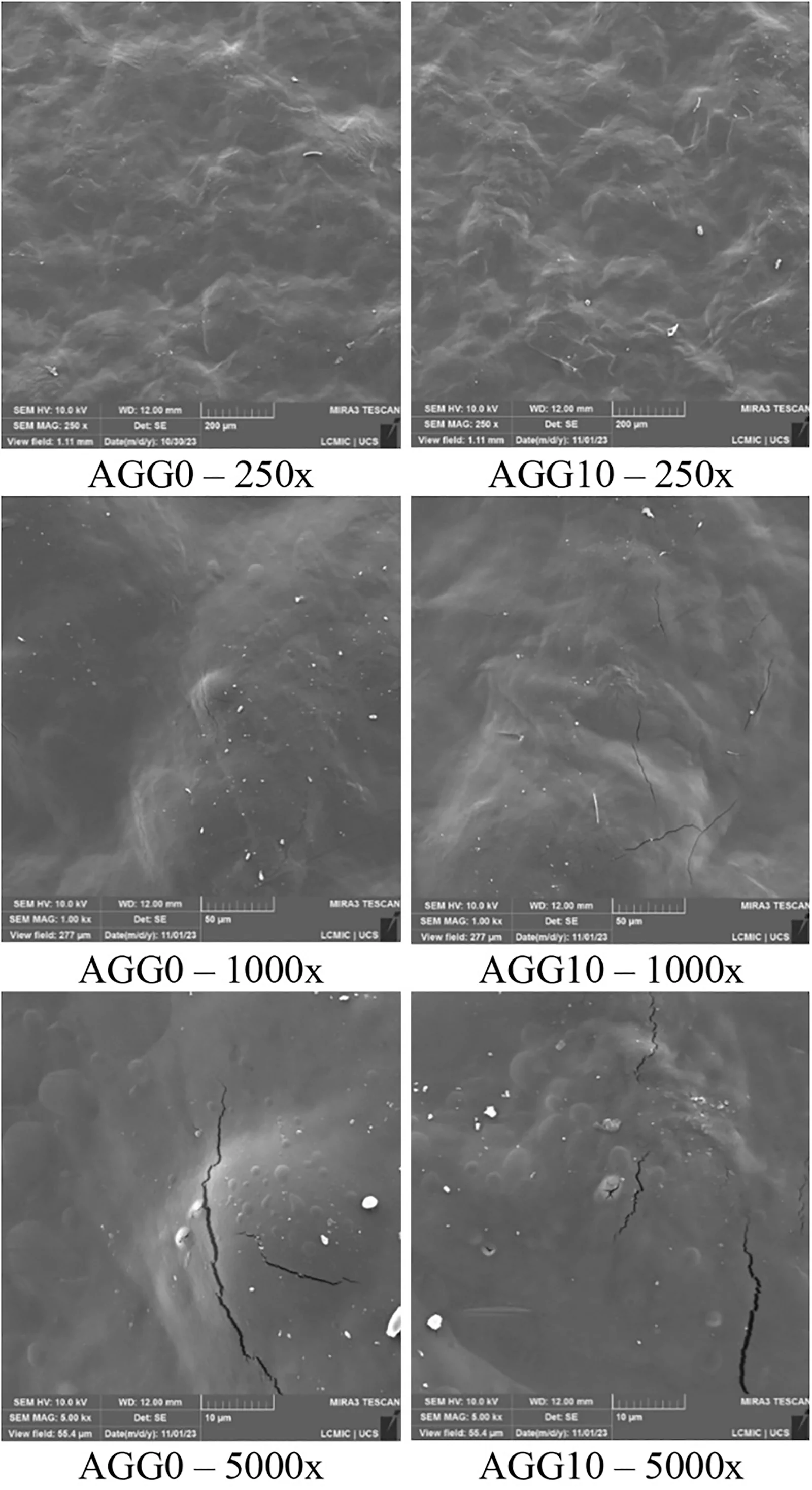
SEM images of the surface of the films AGG0 and AGG10 with magnifications of 250×, 1000×, and 5000×.

At 250× magnification, aspects like those observed in optical microscopy results are observed. The film’s roughness is visible on its surface. Some particles can also be seen over the film; they are attributed to dirt deposited on it due to air drying and storage.

The surface irregularities of the agar films may have resulted from the exudation of glycerol. Glycerol acts as a plasticizer, altering the distance between polymeric chains. As the cross-linking process proceeds, glycerol is displaced by cross-linking bonds, leaving the film’s surface and increasing roughness.^33^

At 1000× magnification, small cracks can be seen. These may have surged due to the drying process. With solvent evaporation and the material contraction due to agar molecule bonding, the resulting stress may cause regions of lower strength on the surface to break, creating cracks and fissures. The cracks are more evident at a 5000× magnification. It is also possible to observe small slopes with blister shapes.

The cross-section of the films, obtained by cryogenic fracturing after freezing with liquid nitrogen, is presented in Figure 5.

**Figure 5:**
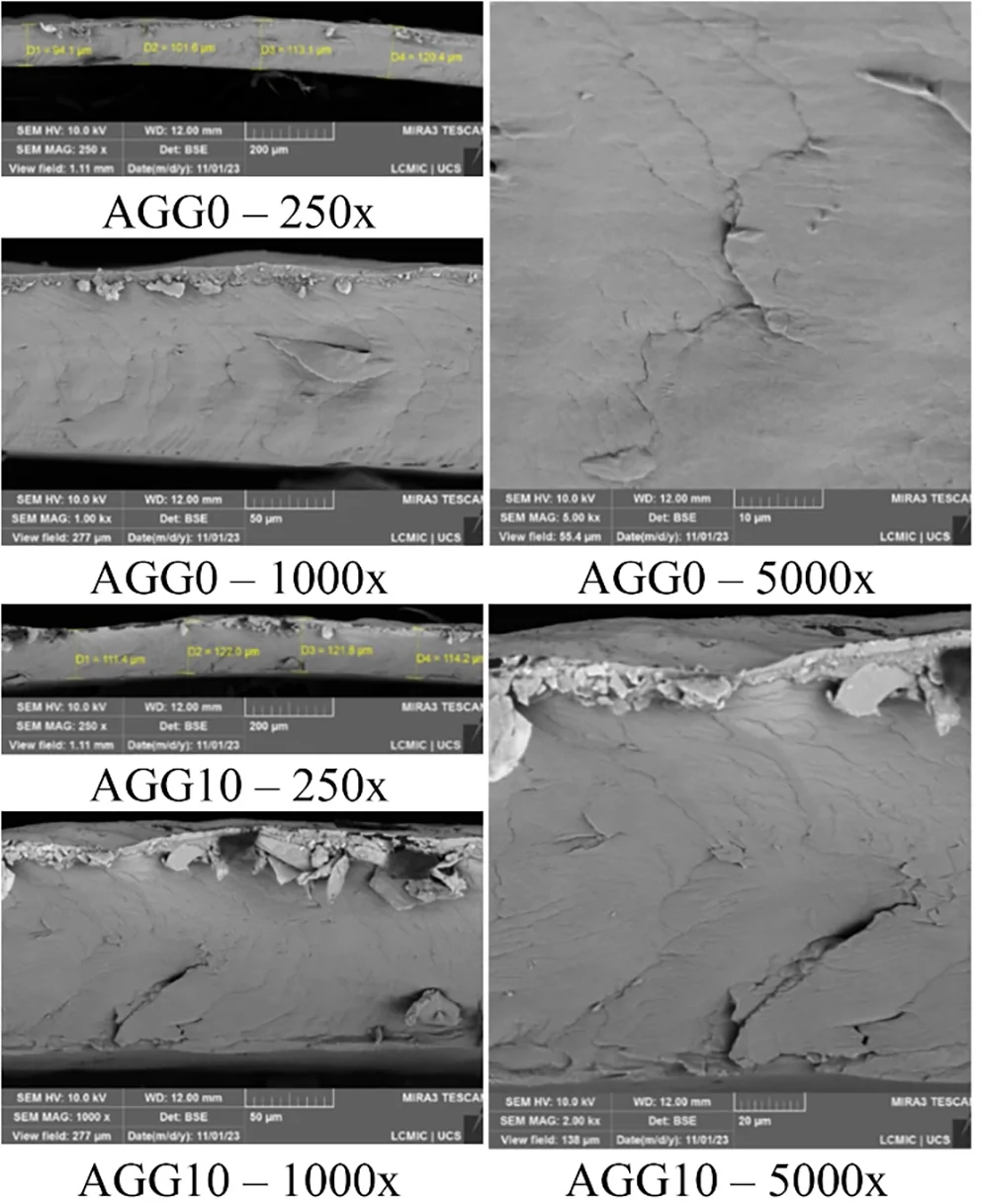
SEM images of the cross-section of the films AGG0 and AGG10, obtained by cryogenic fracturing, with magnifications of 250×, 1000×, and 5000×.

The 250× magnification was also used for thickness measurement. On AGG0 formulation films, the average thickness was 98 ± 13 μm, while the mean thickness on AGG10 formulation was 114 ± 7 μm. The thickness results obtained by the micrographic method are lower than those from film thickness gauge measurements due to differences between the techniques. In SEM microscopic measurements, valleys and peaks of the material’s surface are considered; meanwhile, when using a film thickness gauge, only the peaks are considered, resulting in higher values. It is possible to observe the roughness effects on the film’s surface, which, besides changing the film’s thickness, can also be seen in the secondary plan of the images as peaks and valleys, behind the first plan corresponding to the cross-section.

On 1000× and 5000× magnifications, rupture marks in the material are distributed on transversal lamellar shapes throughout the cross-section. It is observed that particles of small size are close to the material surface. This effect can be attributed to differences in the properties of the surface and the interior of the material. This may also cause differences in the breaking behavior of those regions.

In those images, the material shows complete compaction, as no pores or empty spaces were observed on the interior of the polymer. It was also not seen by any foreign bodies, suggesting high purity of the raw materials and their complete solubilization during cast solution formation.

Agar films commonly present a compact structure. The dense, uniform structure confirms a satisfactory molecular aggregation during solvent evaporation.^33^

### Fourier Transform Infrared Spectroscopy (FTIR)

The Fourier transform infrared (FTIR) spectra for AGG0 and AGG10 formulations and for the raw materials are presented in Figure 6.

**Figure 6:**
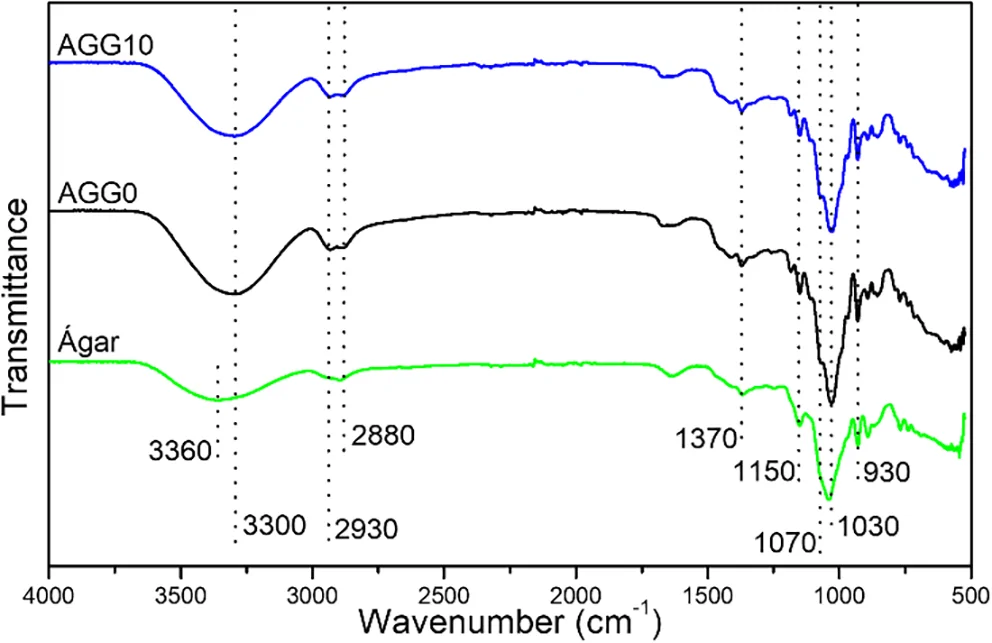
Fourier transform infrared (FTIR) spectra of the AGG0 and AGG10 films and the food-grade agar.

The FTIR spectra show absorption bands at 3300 cm^–1^, corresponding to the O–H bond stretching and at 2880–2930 cm^–1^, corresponding to the C–H bond bending.^34^ The existence of absorption bands on 1150 cm^–1^ and 1370 cm^–1^, corresponding to bending of sulfonyl groups, can also be observed, as well as bands between 1030–1070 cm^–1^, resulting from bending of O–H and C–C bonds.^35^ A specific band at 930 cm^–1^, corresponding to the C–O–C bond vibration of the 3,6-anhydrogalactose bridge can also be observed.^36^

The main difference between the films’ spectra and the agar is the intensity of the 3300 cm^–1^ band, which has its maximum peak at 3360 cm^–1^ in the raw material. Since this band is characteristic of intermolecular bonds in polymers,^34^ it becomes more evident on films, since the polymeric structure is found on them. Besides, in both formulations, glycerol was added, which has hydroxyl groups, contributing to the increase in the intensity of the 3300 cm^–1^ band in the films.

The 1150 cm^–1^ and 1370 cm^–1^ bands, related to the vibration of sulfur compounds, correspond to the agaropectin chains. These chains, with a lower gel strength,^14^ influence agar quality, because the higher the intensity of these bands, the greater the presence of agaropectin and, consequently, the lower the gel strength and quality of the films.^37^

Relevant differences were not observed between the spectra of the films with formulations AGG0 and AGG10, suggesting that any spectral differences may indicate the formation of cross-links. However, as reported by Gönenç and Us,^32^ polysaccharide cross-linking (as agar) with glutaraldehyde would form C–O bonds, which are already present on the structures of both glutaraldehyde and agar. Therefore, these new bonds would be hardly detectable in FTIR spectra. Also, other bands related to glutaraldehyde may have been covered by the other reagents, such as glycerol, that are in higher concentration on the films.

### Tensile Test

The tensile test results of the polymeric films are presented in Table 3.

**Table 3:** Tensile Test Results for the AGG0 and AGG10 Films^a^

Formulation	Tensile Strength (MPa)	Young Modulus (MPa)	Elongation at break (%)
AGG0	16.3 ± 2.6^a^	239 ± 46^a^	55.9 ± 7.8^a^
AGG10	15.4 ± 1.7^a^	220 ± 22^a^	58.3 ± 4.7^a^

a Data presented as mean ± standard deviation. Different letters in the same column indicate statistically different means (*p*-value < 0.05).

Comparing the properties of the produced films with those reported by Hernández et al.,^12^ it can be observed that the films exhibit results compatible with those of other biopolymer and fossil-based polymer films.

Regarding tensile strength, the produced films have properties like those of low-density polyethylene (LDPE), a synthetic polymer with a tensile strength of about 10–20 MPa.^38^ LDPE is used for industrial and commercial packaging, plastic bags, food packaging, agricultural films, and other applications.^39^

AGG0 and AGG10 films also had tensile strength compatible with thermoplastic starch (TPS), which has a tensile strength of 10–50 MPa, and hydroxypropyl cellulose (HPC), with a tensile strength of 12–22 MPa, both biopolymers with potential for practical applications.^12^

Regarding Young Modulus (*E*), the AGG0 and AGG10 formulations shown values higher than those reported by Hernández et al.^12^ Comparing to commercial polymers, once more, the produced material can be compared to LDPE, which has a Young modulus of 130–348 MPa, and polyethylene (PE, 190–240 MPa), with applications similar to those of LDPE and 1,2-polybutylene (PB, 150–295 MPa).^38^

Elongation at break of the produced films aligns with the behavior reported by Hernández et al.^12^ Compared to commercial polymers, elongation is like that of TPS (30–60%) and polypropylene (PP, 10–140%), whose films have the same applications as LDPE. However, PP differs from LDPE and the produced agar polymer in having a higher tensile strength.^38^

Briefly, the synthetic polymer to which the produced films are more similar is LDPE, with differences in strain at break (LDPE has an elongation at break of 130–270%).^38^

No statistical differences were observed between formulations AGG0 and AGG10 (Table 3), indicating that the addition of glutaraldehyde did not influence the properties. However, when analyzing the tensile stress–strain curves presented in Figure 7, it may be seen that the addition of glutaraldehyde resulted in a tendency to lower tensile strength and to enhance the material’s ductility, as evidenced by the increase in strain at break. This behavior indicates that glutaraldehyde may have acted as a plasticizer at the studied concentration. This may have happened due to a weakening of intermolecular bonds, consequently reducing tensile strength and allowing the material to stretch further before breaking.

**Figure 7:**
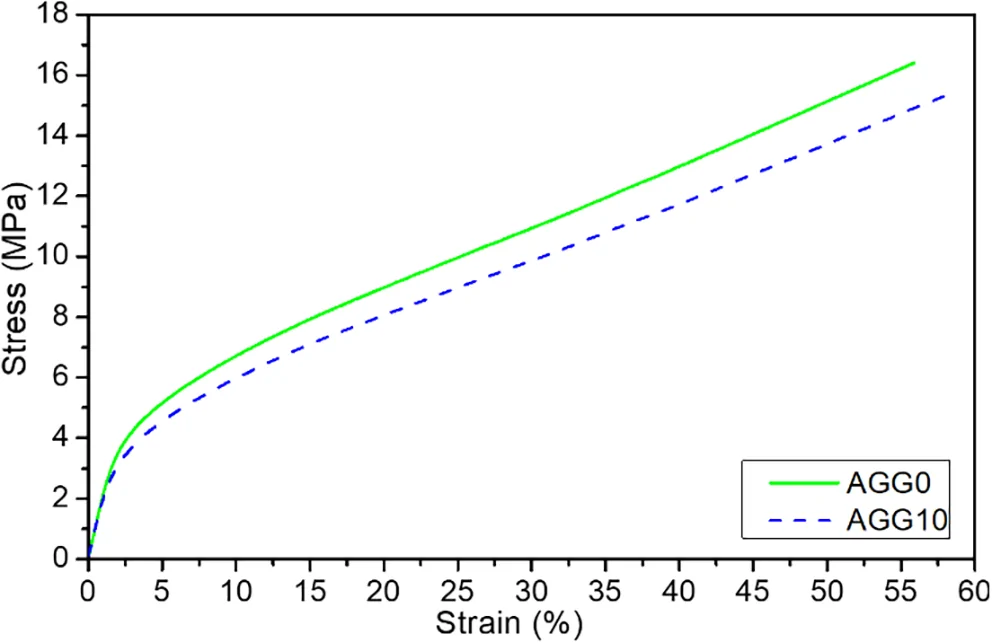
Stress–strain curves of the tensile test for the films AGG0 and AGG10.

The addition of glutaraldehyde was expected to increase tensile strength through cross-linking reactions. However, some additives may cause heterogeneity in the molecular organization of the compound, due to domains of unequal cross-linking degree, forming cross-linked, insoluble regions. Despite sometimes offering advantages when used in polymers, some studies comparing the influence of cross-linking agents in films observe neither differences in properties nor negative effects.^40^

### Ultraviolet–Visible Spectroscopy

The UV–visible absorption spectra of the films produced with and without adding glutaraldehyde are presented in Figure 8.

**Figure 8:**
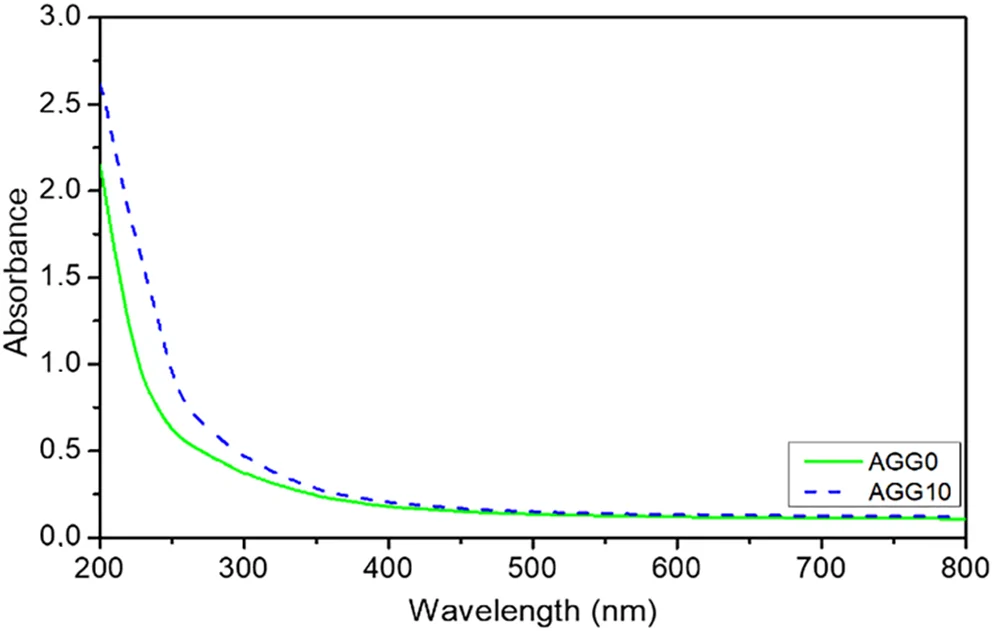
Ultraviolet–visible absorbance spectra of films AGG0 and AGG10.

The curves in Figure 8 exhibit behavior related to the apparent color of the films. In this case, the visualized color is complementary to that of higher absorbance. Because the purple (400–435 nm) wavelength range was most absorbed, the films appeared yellow, with the color intensified in the glutaraldehyde-treated film.

A higher absorbance in the ultraviolet region (200–400 nm) was observed for the films of the AGG10 formulation. This difference, once again, is observed due to the addition of glutaraldehyde, which has a higher absorption in this region. This result aligns with those reported by Scopel et al.,^26^ which demonstrates that adding glutaraldehyde to starch films does not cause cross-linking but enhances absorbance at UV wavelengths.

### Thermogravimetric Analysis

The thermograms obtained from thermogravimetric analysis (TG/DTG) of the films and commercial grade agar are shown in Figure 9, and the quantitative data are described in Table 4.

**Figure 9:**
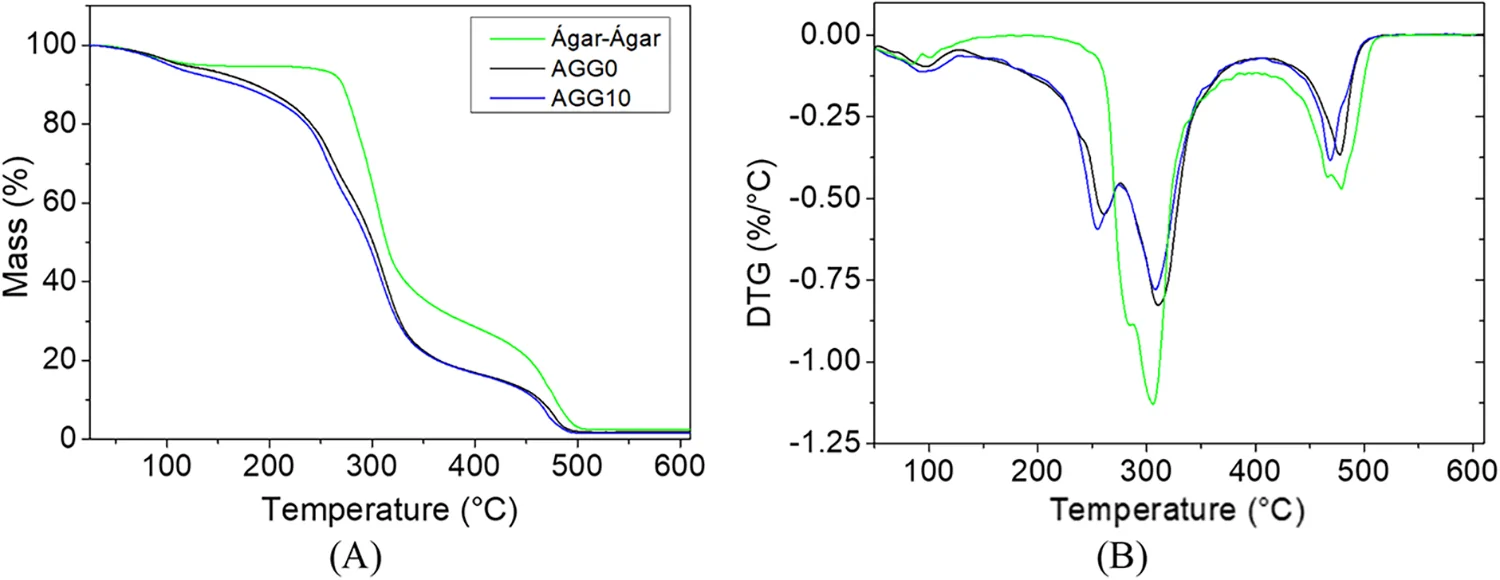
Thermograms (A) and differential thermograms (B) of films AGG0, AGG10, and the food-grade agar.

**Table 4:** Data on the Thermal Behavior of the Films AGG0 and AGG10^a^

Film	Maximum degradation temperature (°C)	Mass loss at first stage (128–275 °C) (%)	Mass loss at second stage (275 – 406 °C) (%)	Mass loss at third stage (406–520 °C) (%)	Residue at 600 °C (%)
Agar	305.8	7.6	59.6	25.4	2.52
AGG0	309.0 ± 1.2 ^a^	30.2 ± 0.2 ^a^	47.6 ± 1.3 ^a^	14.6 ± 0.1 ^a^	1.99 ± 0.65 ^a^
AGG10	309.0 ± 1.6 ^a^	31.9 ± 0.7 ^b^	44.6 ± 3.5 ^a^	14.8 ± 0.7 ^a^	1.58 ± 0.19 ^a^

a Data presented as mean ± standard deviation. Different letters in the same column indicate statistically different means (p-value <0.05). Results for pure agar were not considered in the statistical analysis.

Agar thermogram showed characteristics like those reported by Ouyang et al.^41^ At approximately 100 °C, a small mass loss was observed and attributed to the evaporation of residual moisture. The second, more expressive event, located at 250–300 °C with a peak at 306 °C, is associated with the thermal degradation of the agar constituents.

According to Ouyang et al.,^41^ thermal degradation of agar occurs via an exothermic reaction. Initially, the glycosidic bond is broken (the C–C bond between the chains of β-d-galactose and 3,6-anhydro-α-l-galactose), followed by dehydration (loss of water), and, eventually, by the opening of the cyclic chain, resulting in different substances depending on which point the chain is broken. A last degradation event occurs between 440–500 °C, with a peak at 479 °C. This may be related to the degradation of carbon residues derived from the polymeric chain break of agar.

It is observed that the films exhibit lower thermal stability than agar, as evidenced by a shift in the first degradation event from 250 to 150 °C. This effect is due to the addition of glycerol to the formulations, which generated a peak of mass loss at 260 °C, characteristic of glycerol degradation.^26^ The addition of glycerol has also decreased the bond strength of the polymeric structures, consequently decreasing the gel strength and the mechanical and thermal stability of the films.^20^ This has a direct relation to thermal degradation temperature of the material, which connects the decrease in bond strengths between polymeric chains to the reduction in the polymer’s thermal stability.^41^

## Conclusions

In this work, agar polymeric films were produced by adding glycerol and glutaraldehyde to their formulation. The produced films were continuous and homogeneous and exhibited visual and sensorial characteristics like those of polymeric films used in general packaging, regardless of the formulation. Adding glutaraldehyde altered the surface properties of the films, decreasing their interactions with water. The formulations AGG0 and AGG10 showed similar physical-chemical properties, showing that glutaraldehyde did not influence the properties of the formulation. Based on the results, it is not possible to confirm cross-linking of agar chains using glutaraldehyde. The production and characterization of agar-based polymeric films demonstrate their potential as a raw material for developing biopolymers as alternatives to fossil-based polymers, thereby strengthening the sustainability of this product. It is important to note that the films with the obtained characteristics, apart from their formulation, showed potential for use as packaging, necessitating a compatibility assessment for each specific application, especially regarding the exposure to moisture and wet environments, since swelling and solubility are higher than those found in fossil-based polymers. Although glutaraldehyde is a widely used cross-linking agent, its application in food-contact materials requires careful control of residual aldehyde because unreacted glutaraldehyde may raise toxicity concerns. Since effective cross-linking was not confirmed in the present study, further investigations should include residual glutaraldehyde quantification, biodegradation studies, barrier properties, and life-cycle assessment before practical packaging applications can be proposed.

## Data Availability

All data produced in this research is shown in the article.
